# Tribology of Pore-Textured Hard Surfaces under Physiological Conditions: Effects of Texture Scales

**DOI:** 10.1021/acs.langmuir.2c03377

**Published:** 2023-05-01

**Authors:** Yiwen Xi, Chang-Hwan Choi, Robert Chang, Hans Jan Kaper, Prashant Kumar Sharma

**Affiliations:** † Department of Mechanical Engineering, Stevens Institute of Technology, Castle Point on Hudson, Hoboken, New Jersey 07030, United States; ‡ Department of Biomedical Engineering-FB40, University of Groningen and University Medical Center Groningen, A. Deusinglaan 1, Groningen 9713 AV, The Netherlands; § University of Groningen, University Medical Center Groningen, W.J. Kolff Institute for Biomedical Engineering and Materials Science-FB41, A. Deusinglaan 1, Groningen 9713 AV, The Netherlands

## Abstract

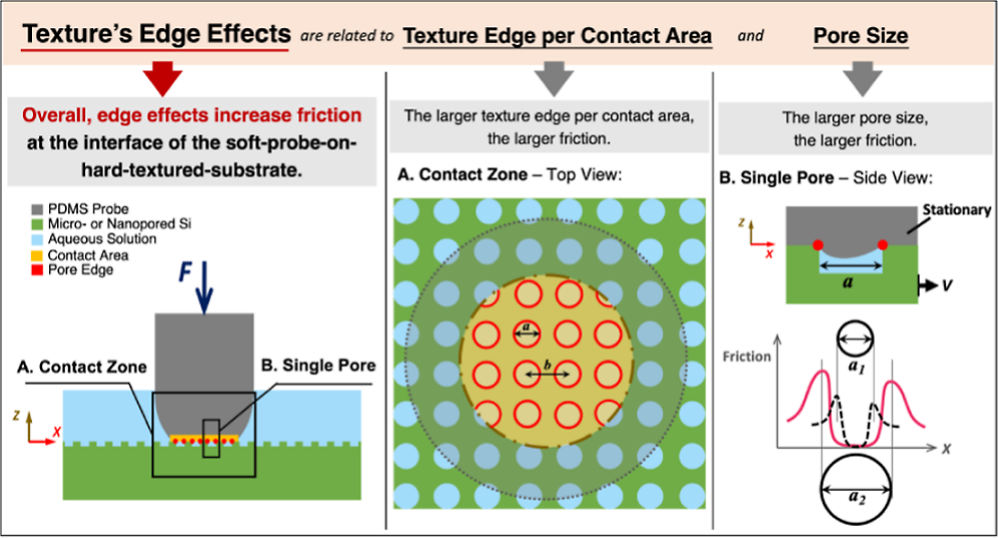

Micro- and nanotexturing on hard biomaterials have shown advantages for tissue engineering and antifouling applications. However, a growing number of studies have also shown that texturing may cause an increase in friction, demanding further research on the tribological effects of texturing under physiological conditions. This study investigates the tribological effects of micro- and nanopore patterns on hard hydrophilic silicon sliding against soft hydrophobic polydimethylsiloxane (PDMS) immersed in aqueous liquids with various viscosities, simulating the sliding of a textured implant surface against soft tissues. The experimental results show that silicon surfaces with pore textures at both micro- and nanoscale feature sizes confer a higher coefficient of friction (COF) than an untextured one. It is attributed to the texture’s edge effect caused by the periodic pore patterns between the two sliding objects with a large difference in material stiffness. For the same solid area fraction, nanopored surfaces show a higher COF than micropored surfaces because of the significantly higher texture edge length per unit area. For micropored surfaces with a similar length of texture edge length per unit area, the COF increases more significantly with the increase in pore size because of the greater stress at the rims of the larger pores. The COFs of both micro- and nanoscale pores generally decrease from ∼10 to 0.1 with an increase in the surrounding aqueous viscosity, indicating the transition from a boundary lubrication to a mixed lubrication regime while mostly remaining in boundary lubrication. In contrast, the COF of an untextured surface decreases from ∼1 to 0.01, indicating that it mostly remains in the mixed lubrication regime while showing the tendency toward hydrodynamic lubrication. Compared to a hydrophilic hard probe sliding against a textured hydrophobic soft substrate, the hydrophobic soft probe sliding against a textured hydrophilic hard substrate produces a significantly higher COF under similar physiological conditions due to the larger edge effect.

## 1 Introduction

Micro- and nanotexturing have broadly been applied to biomaterial implant surfaces to enhance cell and tissue biological function, including neural outgrowth, bone repair, and tissue regeneration, along with the material’s antifouling performance to reduce the continuous risk of infection.^1−4^ However, upon implantation within the human body, the textured surfaces will invariably slide against tissues and organs. This is particularly evident at the musculoskeletal joints or in locations where frequent movement between tissue pairings is involved, such as the eyelid and eyeball, between tongue, mucosa, and teeth surfaces in the oral cavity, or soft tissue against percutaneous pins used for external fixator frames.

Recently, several studies have shown that textured surfaces can result in a significant increase in friction, measured in terms of coefficient of friction (COF) or dissipated frictional energy, depending on the tribological conditions, such as sliding speed, applied normal load, and the mechanical properties of the paired surfaces, such as material stiffness, hydrophobicity, and water content.^5−8^ In one study, the incorporation of microscale pore textures on a hydrophobic soft material of polydimethylsiloxane (PDMS) was shown to generally increase the friction in sliding against a hydrophilic hard material of glass.^5^ In contrast, the outcomes from another study indicate that the micropore textures on a hydrophilic hydrogel material of poly(2-hydroxyethyl methacrylate) (pHEMA) reduce the friction in sliding against the glass.^9^ Based on the apparent antagonistic effects of the micropore textures on friction, these two studies collectively suggest that the state of the aqueous liquid configured at the interface during sliding should play an important role in the tribological properties. Specifically, the extent of the aqueous liquid environment effect will depend on the wettability and softness of the textured surface as well as the availability and absorptivity of water at the contact interface. Hence, it is critical to understand the tribological properties of the textured surfaces within the context of a physiologically relevant aqueous environment. Clinically, high friction as rendered by textured surfaces can cause undesirable mechanical wear of human tissue, increasing the risk of infection and inflammation and gradually leading to severe or permanent injuries, such as corneal blindness and chronic osteoarthritis.^10,11^

An important aspect of soft tissue sliding against textured, hard biomaterials is the large stiffness difference. Human soft tissue varies in stiffness from around a few hundred pascals (brain) to a few tens of kilopascals (muscle), which is 10^6^ to 10^9^ times softer than the hard biomaterials used for implants, such as stainless steel, CoCrMo, Ti6AL4V, alumina, and yttria-stabilized zirconia.^12^ This pronounced difference in stiffness values allows for significant deformation of the much softer tissue. Hitherto, the tribological effects of micropore (also known as micro-dimple) textures on paired hard materials have been reported for machine bearing applications where both stiff materials contact and slide past each other at high unidirectional sliding speeds with no or little deformation.^13−15^ Furthermore, the friction generated inside the human body is dependent on the environment around the two contact surfaces. For example, the viscosity of the fluid involved in human movement can be varied from a few mPa·s to 10^4^ mPa·s (i.e., the viscosity of blood in a vein is 1.2 mPa·s,^16^ and the viscosity of the synovial fluid is from 10^3^ to 10^4^ mPa·s.^17^ However, little is understood about the tribological behaviors of micropored hard materials sliding against soft materials under physiologically relevant conditions, such as reciprocating in a delicate environment with relatively low contact pressure (in kPa) and sliding speed (in mm/s) in an aqueous environment with various viscosities. Moreover, there is even less literature reporting on the tribological effects of nanoscale pore textures compared to their microscale counterparts.

Based on the identified knowledge gaps, in this study, we investigate the tribological effects of both the micro- and nanoscale pore textures of hydrophilic hard material of silicon (Si) sliding against hydrophobic soft material of PDMS are investigated under physiologically relevant conditions to simulate the tribological conditions between a pore-textured implant surface of hard material and soft tissue. This pair also allows for the comparison to the previous work, where the fundamentally same materials (i.e., glass is comparable to silicon in terms of both mechanical properties and wettability) were used while the micropore textures were implemented onto the soft PDMS material instead of the hard glass material.^5^ In this investigation, the COF values of the micro- and nanopored surfaces of silicon are measured and compared to those of an untextured surface. The solid area fractions (ϕ) of the surfaces are varied, ranging from 1 (corresponding to the untextured surface) to 0.8 (corresponding to the largest pore diameter at the given pattern periodicity). The viscosities of the surrounding aqueous solution are varied from 1 to 100 mPa·s to mimic the different biofluids in the human body. While the contact pressure is fixed at 100 kPa, the sliding velocity is varied from 0.05 to 2 mm/s in the reciprocating sliding mode to mimic physiologically relevant conditions such as blinking, chewing, and walking.

## 2 Materials and Methods

### 2.1 Micro- and Nanopored Silicon Surfaces

The periodic microscale and nanoscale pore patterns were first registered onto the photoresist layer spin-coated onto silicon wafers by using photolithography and laser interference lithography, respectively.

As for the silicon wafers, 4-inch single-side polished (surface roughness, *R*_a_ < 5 nm) silicon wafers (orientation <100>, P type) were purchased from University Wafer (South Boston, MA, USA). Before lithography, each wafer was cleaned with acetone, ethanol, and deionized water, followed by blow-drying in nitrogen (N_2_) gas and dehydration on a hotplate at 180 °C for 5 min.

As for the photolithography, hexamethyldisilazane (HMDS, Sigma-Aldrich, St. Louis, MO, USA), as an adhesion promoter, was first spin-coated (PWM32, Headway Research, Garland, TX, USA) on top of the polished side of each silicon wafer at 3000 rpm for 1 min. Then, a layer of positive photoresist (SPR 3012, Shipley L.L.C.-Rohm and Haas Electronic Materials, Marlborough, MA, USA) was spin-coated onto the layer of HMDS at 2000 rpm for 1 min, followed by a soft baking on a hotplate at 95 °C for 1 min. The photoresist layer was exposed to ultraviolet (UV, wavelength ∼250 nm) radiation through a photomask of periodic pore patterns by using a mask aligner (MA6, Suss Micro Tec SE, Germany), followed by a hard-baking on a hotplate at 115 °C for 1 min. For the development of the photoresist layer, each wafer was then fully immersed in a developing solution (MF-319, Shipley L.L.C.-Rohm and Haas Electronic Materials, Marlborough, MA, USA) at room temperature for 1 min, followed by rinsing with deionized water and blow-drying with nitrogen gas.

As for the laser interference lithography, silicon wafers were cut into square pieces of 30 × 30 mm^2^ with a diamond-tip scriber (Techni-Pro, Worcester, PA, USA) to ensure uniform patterns over the coverage area.^18^ A positive photoresist (PR1-2000A, Futurrex Inc., Franklin, NJ, USA) diluted in a solvent (SD1, Futurrex Inc., Franklin, NJ, USA) in a volume ratio of 1:10 was spin-coated on the polished side of the cut pieces at 6000 rpm for 30 s to have a film thickness of ∼50 nm, followed by a soft-bake on a hotplate at 115 °C for 1 min. The photoresist layer was then exposed to register a square array of periodic pore patterns by using the Lloyd-mirror interference lithography setup consisting of the He-Cd laser (IK3501RG, Kimmon Electric, Japan) of 50 mW and 30 cm in coherence length at 325 nm in wavelength,^18,19^ followed by the development in a RD6 solution (Futurrex Inc., Franklin, NJ, USA) diluted with deionized water in the volume ratio of 1:1 for 10 s. The size of the developed nanopore was mainly dependent on the energy dose (mJ/cm^2^), i.e., the exposure time in the exposure step. After the development, the pieces were rinsed in deionized water for 30 s and blow-dried with nitrogen gas.

After the lithography steps, the pore patterns created on the photoresist layer were transferred to the underlying silicon substrates by using deep reactive ion etching (DRIE, Oxford Plasmalab 100, Oxford Instruments, UK),^19,20^ where the depth of the pore patterns transferred to the silicon substrates was dependent on the etching time. The photoresist layer served as the etch mask layer in the DRIE process was then removed by Piranha solution (H_2_SO_4_/H_2_O_2_ = 3:1 in volume), followed by rinsing in deionized water and blow-drying with nitrogen gas. The contact angle of a sessile droplet of water on the nanopored silicon surfaces was less than 10°, indicating the hydrophilicity of the surfaces.

**Figure 1:**
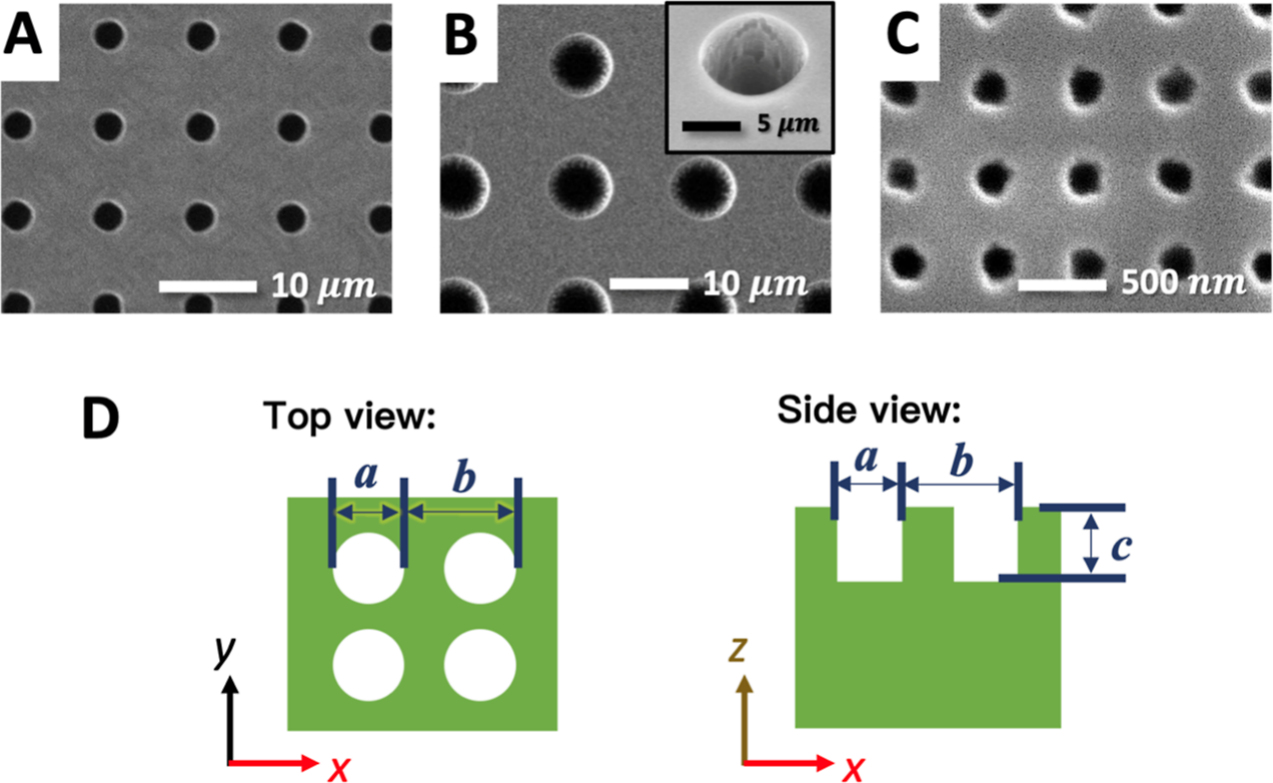
(A–C) SEM top-view images of the pored silicon surfaces. The inset in (B) shows the enlarged, angled view of the surface. (D) Schematic representation of the pore dimensions.

Figure 1 shows the scanning electron microscopy (SEM) images of the micropored (Figure 1A,B) and nanopored (Figure 1C) silicon surfaces. For the measurement of tribology properties, the silicon substrates were cut into a square piece of 10 × 10 mm^2^. The dimensions (i.e., pore diameter, pattern periodicity, and pore depth) of the pore textures are schematically represented in Figure 1D and summarized in Table 1. An untextured silicon wafer (ϕ = 1) was used as a control for comparison to the pored surfaces. The three pore-textured surfaces are named by their texture scales and solid area fractions as follows: nano ϕ = 0.9, micro ϕ = 0.9, and micro ϕ = 0.8.

**Table 1:** Dimensions of the Pore Patterns, Where ϕ is a Solid Area Fraction Defined as

texture type			diameter, *a*	period, *b*	depth, *c*
untextured	ϕ = 1		N/A	N/A	N/A
micro	ϕ = 0.92 ≈ 0.9	(Figure 1A)	3.6 μm	10.0 μm	∼10 μm
micro	ϕ = 0.76 ≈ 0.8	(Figure 1B)	8.0 μm	15.0 μm	∼10 μm
nano	ϕ = 0.88 ≈ 0.9	(Figure 1C)	200 nm	500 nm	∼200 nm

### 2.2 PDMS Probe

A hemispherical PDMS probe was prepared as the tribological pair for the silicon surfaces by using a molding technique. The PDMS mixture (monomer/cure agent = 10:1 in volume, Sylgard-184, Dow Corning, Midland, MI, USA) was first poured into a single well of a 96-well (round bottom) clear polystyrene (PS) microplate (Greiner Bio-One, Frickenhausen, Germany) and cured at 80 °C overnight (>12 h) in a vacuum chamber. The cured probe was then gently pulled out of the microplate. The radius and length of the cured PDMS probe were measured to be 3.1 and 10 mm, respectively, as defined by the dimensions of the microplate well. After demolding, *n*-hexane (Sigma-Aldrich, St. Louis, MO, USA) was used to remove dust and fingerprints from the PDMS surfaces. After quickly soaking in the *n*-hexane, the PDMS probes were immediately rinsed with Milli-Q water and sonicated in Milli-Q water for 15 min. The roughness of the PDMS probe (*R*_a_) is determined by a microplate well, which was ∼5 nm.

### 2.3 Tribological Experiments

**Figure 2:**
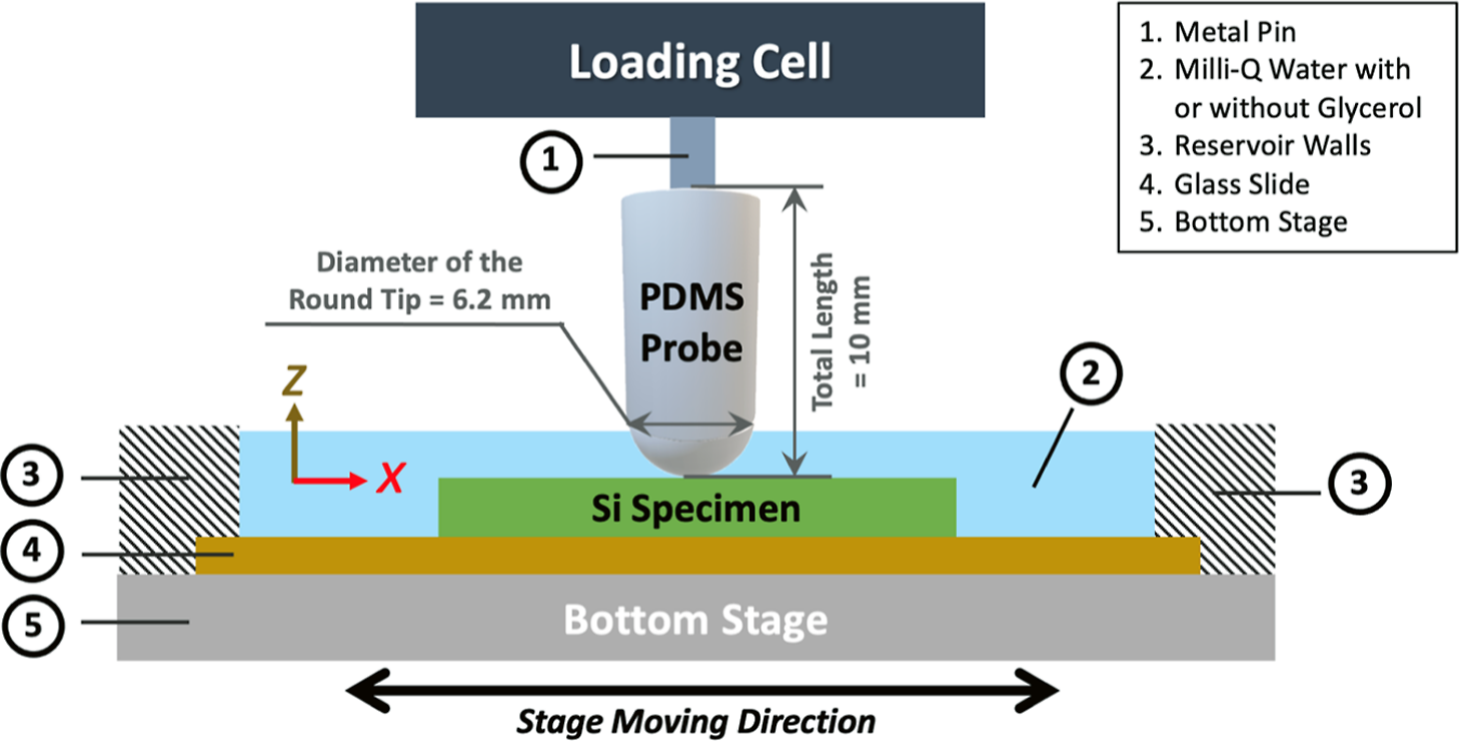
Schematic, drawn not to scale, of the measurement of the COF during the tribological experiments.

The COF values of the prepared silicon surfaces were measured using a Universal Micro Tribometer (UMT-3, Bruker, Billerica, MA, USA) in a reciprocating sliding mode (Figure 2). To fix the PDMS probe onto the UMT-3 loading cell having a small mount, a thin metal pin (0.6 mm in diameter and 15 mm in length) was partially inserted into each PDMS probe (∼5 mm deep down) through the flat top of the probe. Each silicon specimen was attached to a glass slide (25 mm × 75 mm) by epoxy glue. The glass slide was then mounted on the bottom of a stainless-steel reservoir, which was placed on the lower stage of the tribometer that can move to generate various sliding speeds. The size of the stainless-steel was slightly larger than that of the glass slide and has the depth of ∼3 mm to hold the aqueous solution. In this study, each specimen of the silicon substrate was slid against the hemispherical PDMS probe at a fixed contact pressure of 100 kPa at varying speeds (*V* = 0.05, 0.1, 0.2, 0.5, 1, and 2 mm/s) in the aqueous solution of glycerol with various viscosities (η = 1, 3, 10, 30, and 100 mPa·s, modified by mixing different proportions of glycerol with Milli-Q water) at room temperature (∼25 °C). The total sliding distance in each tribological test was 40 mm (2 mm × 2 directions × 10 cycles). The COF values, measured by the tribometer with a data sampling frequency of 1000 Hz, were used to evaluate the tribological properties. Of note, only the data in the aqueous solutions of 3 and 30 mPa·s were collected and used for the surface of micro ϕ = 0.8 because the data measured under the other viscosities go beyond the upper limit of the loading sensor of the instrument. The raw data were analyzed by a MATLAB script, which extracted a mean COF value for each sliding cycle. An average COF value of the mean value of the middle eight cycles (i.e., discarding the first and last cycles showing the unsteady behaviors due to the acceleration and deceleration in the sliding speed with a destabilized load at the beginning and end of each test) was then calculated. Each experiment was repeated three times under the same conditions. Then, the average of the three experiments (i.e., the mean COF value of a total of 24 cycles for the middle 8 cycles with 3 times) for the given condition was finally used for the statistical analysis. For the statistical analysis, the Student’s *t*-test and two-way ANOVA with Tukey’s multiple comparisons test were used. A significant difference was assumed for *p* < 0.05.

### 2.4 Distribution of Pore Textures at the Contact Zone

To achieve the same average contact pressure (*P* = 100 kPa) for the specimens, different loads (*F*, in mN) were applied on the silicon surfaces depending on their solid area fractions (ϕ), based on the Hertzian contact theory. For the given contact pressure, *P*, over the compressed PDMS interface by the silicon substrate, the applied initial normal load, *F*, is estimated by
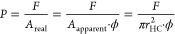
1where *A*_real_ is the real contact area at the interface, *A*_apparent_ is the apparent area at the contact zone (which includes the real contact area), ϕ is the solid area fraction of the silicon surface, and *r*_HC_ is the contact radius of a PDMS on the silicon substrate. According to the Hertzian contact theory, *r*_HC_ is obtained as
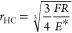
2where *R* is the radius of the PDMS probe (3.1 mm). E* is the interfacial stiffness, which can be calculated by
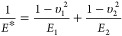
3where *E*_1_ and *E*_2_ and υ_1_ and υ_2_ are the Young’s moduli and Poisson’s ratios for the PDMS probe (subscript 1) and silicon substrate (subscript 2), respectively. Those values are summarized in Table S1 in the Supporting Information. Of note, although PDMS was tested and reported as a viscoelastic material in some studies, our PDMS probe can be considered a highly elastic solid material under the test conditions (i.e., temperature and sliding speed) in this study.^21−23^ Hence, a constant value is used as the Young’s modulus of PDMS for the Hertzian contact theory. The contact radii estimated for the tribological experiment conditions based on the Hertzian contact theory are also summarized in Table S2 in the Supporting Information.

Based on the calculation results of the apparent contact area for each silicon specimen, the number of pores, *n*, in the apparent contact area can be calculated as
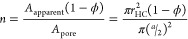
4where *a* is the diameter of a pore (see Table 1). The circumference of each pore, *C*_pore_, equals π*a*. The total length of texture edge inside the apparent contact area (*L*_total_), as shown in Figure 4, is then equal to *C*_pore_ × *n*. The ratio of the texture edge length per unit area (*Q*) can then be estimated by *C*_pore_ × *n*/*A*_apparent_, summarized in Table 2.

**Table 2:** Distribution-Related Variables of the Pore Textures at the Apparent Contact Area

solid area fraction, ϕ	number of pores in the contact area, *n*	total length of texture edge in the contact area, *L*_total_ (μm)	ratio of texture edge length per unit area, *Q* (μm/μm^2^)
1 (untextured)	0	0	0
0.9 (micro)	1.11 × 10^3^	1.26 × 10^4^	0.11
0.8 (micro)	3.60 × 10^2^	8.97 × 10^3^	0.10
0.9 (nano)	3.60 × 10^5^	2.26 × 10^5^	2.00

## 3 Results and Discussion

### 3.1 Effects on the COF

**Figure 3:**
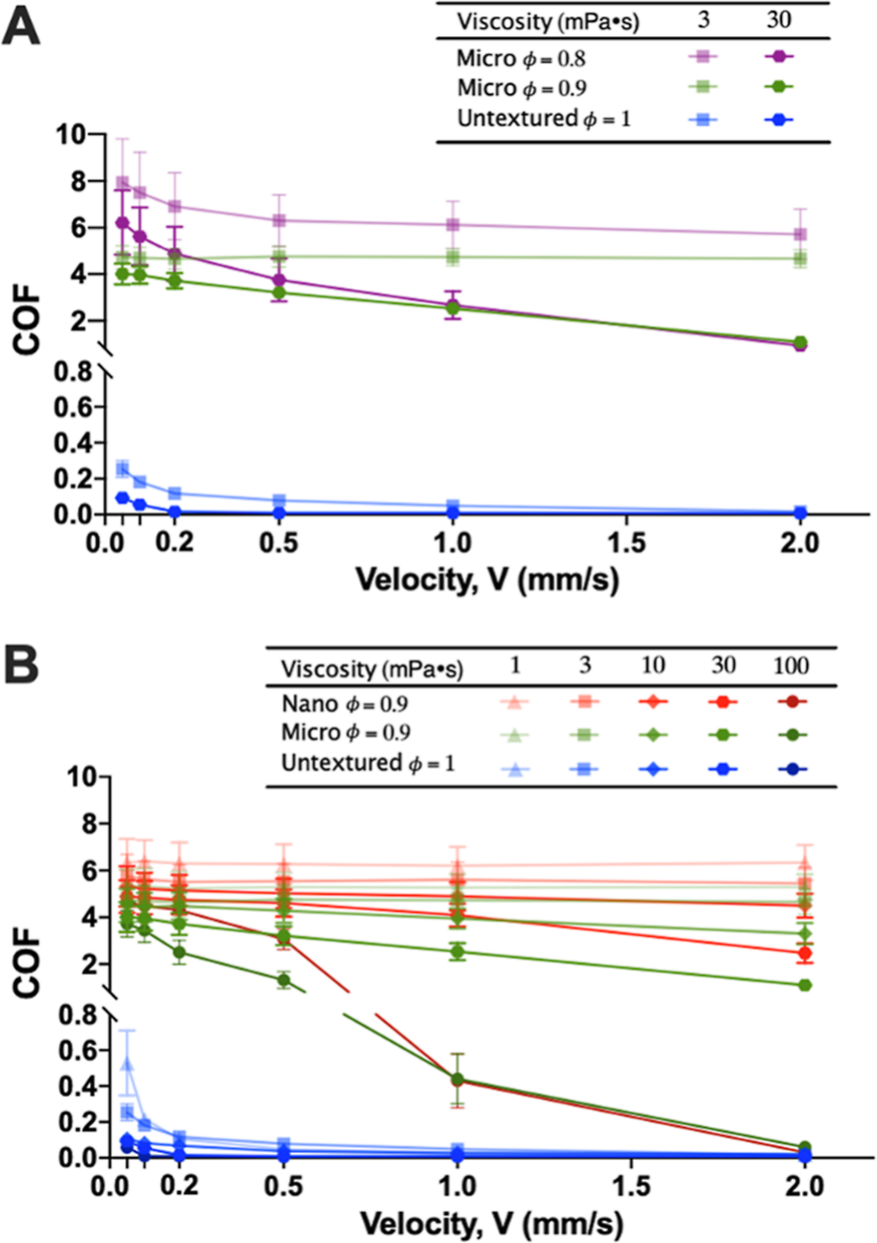
Mean COF value with respect to the sliding speed under the constant contact pressure of 100 kPa and various aqueous viscosities of 1–100 mPa·s: (A) micropored surfaces (micro ϕ = 0.8 and micro ϕ = 0.9) compared to the untextured surface (ϕ = 1); (B) nanopored surface (nano ϕ = 0.9) compared to the micropored surface with the same solid area fraction (micro ϕ = 0.9) and also the untextured surface Si (ϕ = 1). The error bars in the graphs represent standard deviations. See Tables S3 and S4 in the Supporting Information for the results of the two-way ANOVA analysis.

Overall, despite some exceptions, the experimental result indicates that both micro- and nanoscale pore textures on the silicon surface increase the COF significantly (*p* < 0.05; see Figure 3 and Table S3 in the Supporting Information) compared to the untextured surface under the same contact pressure, sliding speed, and aqueous viscosity. Specifically, Figure 3A shows the COF values of the micropored surfaces compared to the untextured surface. At the aqueous viscosity ranging from 3 to 30 mPa·s, the COF values show the following trend: COF_micro ϕ = 0.8_ > COF_micro ϕ = 0.9_ > COF_ϕ = 1_ (with *p* < 0.05; see Table S3). The result suggests that the micropore texture generally increases the COF, which should be more pronounced with the decrease in the solid area fraction, ϕ. Meanwhile, Figure 3B shows the COF values of the nanopored surface compared to those of the micropored surface with the same solid area fraction and the untextured surface. At an aqueous viscosity ranging from 1 to 100 mPa·s, the COF values show the following trend: COF_nano ϕ = 0.9_ > COF_micro ϕ = 0.9_ > COF_ϕ = 1_ in most cases (with *p* < 0.0005; see Table S3). The exception is only for the case of nano ϕ = 0.9 *vs* ϕ = 1 with the fastest sliding speed of 2 mm/s under the most viscous aqueous solution of 100 mPa·s (*p* > 0.99) and for the case of nano ϕ = 0.9 *vs* micro ϕ = 0.9 with the sliding speeds of 1 and 2 mm/s under the most viscous aqueous solution of 100 mPa·s (*p* > 0.8), where the pored surfaces do not show any significant difference to the untextured surface.

**Figure 4:**
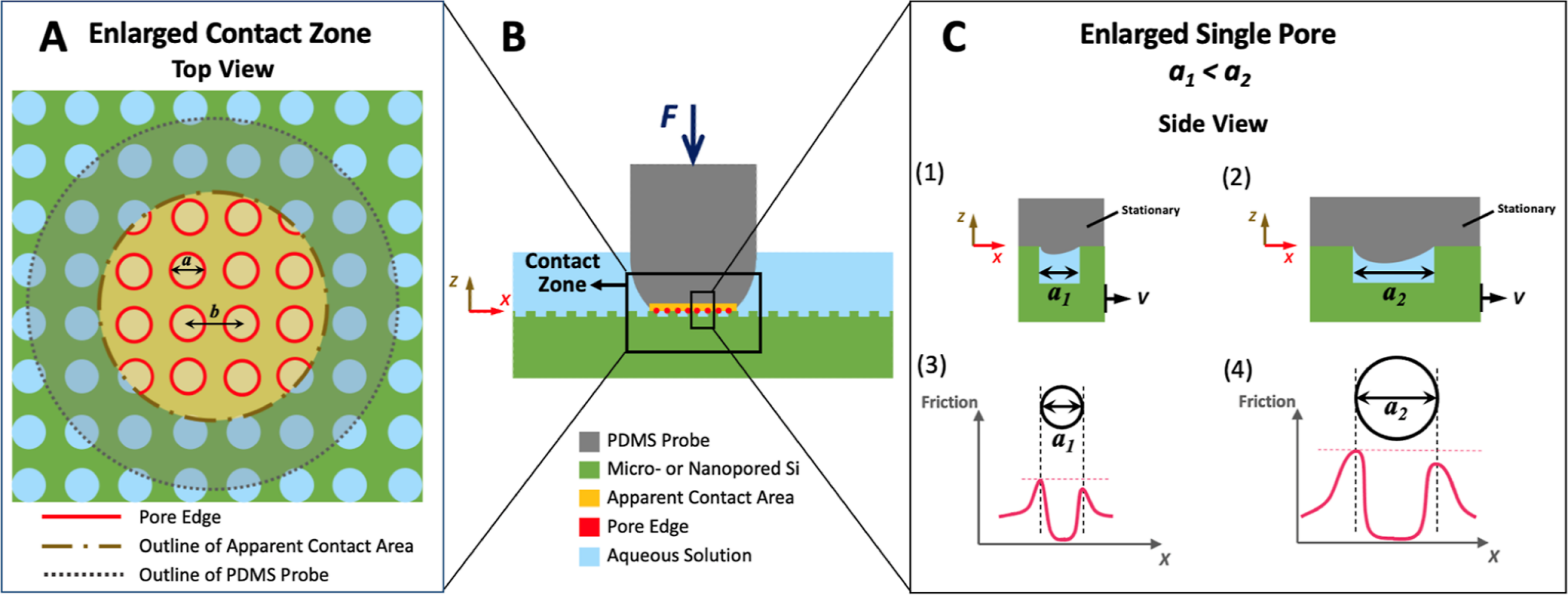
Texture’s edge effect on pore patterns (drawn not to scale): (A) schematic of the texture edges in the contact area; (B) schematic of the contact interface between the tribo-paired surfaces; (C) schematic of the contact interface for a single pore with a different pore size (diameter: *a*_1_ < *a*_2_) and the corresponding friction (not to real scale) along the interface. Higher friction is expected at the leading edge than at the trailing edge, which needs further experimental or theoretical proof in future studies.

The increase in the COF by the micro- and nanopore textures is attributed to the texture’s edge effect at the interface, where the soft PDMS probe slides against the edges of the pores of the hard silicon surface, as schematically illustrated in Figure 4A. In this study, the texture’s edge effect, as a phenomenon occurring at the heterogeneous contact interface due to the pore patterns leading to the increase in friction, can be explained by the two different aspects. One is the large difference in material stiffness (i.e., ∼5 × 10^4^ times difference in Young’s moduli) between the PDMS probe and the silicon substrate. The other is the morphology (i.e., shape and dimension) of the surface pattern. As schematically illustrated in Figure 4B, under normal load (*F*), the soft PDMS probe is expected to be flattened by the hard silicon substrate at the contact zone. Meanwhile, the PDMS placed over the pores would further deform downward (i.e., partially penetrate the pores), as schematically illustrated in Figure 4C. During the sliding movement, the stress induced by the deformation of the soft PDMS probe should concentrate and rise across the edges of the hard silicon pores. Such accumulated stress from the deformation of the viscoelastic PDMS at the edges will produce large frictional energy and enhance the friction, resulting in a higher COF value than that on a nontextured smooth surface with a homogeneous contact interface and aggravating the potential for wear and large energy dissipation at the interface.^24−31^ This study shows a significant difference in COF between the textured and untextured surfaces, especially at relatively low sliding speeds where discontinuous jumps (i.e., “snap-offs” due to the partial penetration into the pores) and local deformation (due to the shear) of the viscoelastic PDMS could occur simultaneously. The edge effect from the textures exacerbates the potential for wear and significant energy dissipation at the interface. Such edge effects are expected to be more pronounced for the pore patterns with the longer texture edge length in the contact area or the pore patterns with a larger pore diameter (*a*).

To understand the edge effect for varied pore dimensions (i.e., pore diameter and period) on the friction, we introduce a new variable, *Q*, which is defined as the ratio of the total texture length of edges in the contact area to the apparent contact area, called the “texture edge length per unit area” (μm/μm^2^). As summarized in Table 2, the ratio *Q* of the nanopored surface (nano ϕ = 0.9) is almost 20 times that of the micropored surface (micro ϕ = 0.9) with the same solid area fraction under the same contact pressure. Although the local increase in friction across the micropore edge would be greater than that across the nanopore edge because of the larger pore size and deformation of the soft PDMS probe, the number of pores and the total contact length of the edges per unit area are much greater for the nanopored surface than the micropored surface. Thus, despite the same solid area fraction, the nanopored surface shows a higher COF (1.2 to 2.3 times in terms of the mean COF value) than the micropored surface in most cases in our study, agreeing with the expectation.

However, the texture edge length per unit area (*Q*) should not be recognized as the only aspect of the texture’s edge effect. It should be noted that the surface of micro ϕ = 0.8 shows higher COF values than the surface of micro ϕ = 0.9, while the ratio (*Q*) of micro ϕ = 0.8 is almost the same as that of micro ϕ = 0.9 (see Table 2). Meanwhile, it should also be noted that the pore diameter of micro ϕ = 0.8 is twice greater than that of micro ϕ = 0.9 (see Table 1). As illustrated in Figure 4C, the larger deformation of the soft PDMS probe into the larger diameter pore can induce a greater stress at the pore edges, significantly increasing the friction on the micropored surface. Thus, the texture’s edge effect for the aspect of the pore size should be more pronounced on micro ϕ = 0.8 than that of micro ϕ = 0.9.

The trend shown in the comparison between micro ϕ = 0.8 and micro ϕ = 0.9 agrees with other reports^9,30−33^ They showed that when a surface was textured with a periodic pattern with a larger feature size of the non-solid area, the greater stress at the edges would make the contact interface more unstable and result in higher friction at the texture edges in the contact area. Yet, the effect of the texture or edge size and density on friction cannot simply be predicted or generalized by the geometric factors because the tribological properties of the materials are also dependent on the other parameters of the tribological system, such as paired materials, the radius of the paired interface, sliding speed, and applied load.^34^ In this study, the texture’s edge effects in both aspects, including the texture edge length per unit area (*Q*) and the individual pore size, appear critical to the friction because of the large difference in material stiffness between the two tribo-paired surfaces. A single property of one material or the contact condition cannot simply determine the friction for the entire tribo-system.

Moreover, unlike a tribological system under a dry condition (i.e., in air), a tribological system under a wet condition makes the situation at the interface far more complex than a simple solid-solid contact. The morphology of micro- and nanotextured surfaces was found to affect the formation of the interfacial liquid film during sliding as well as the transition of lubrication regimes when the solution viscosity and sliding speed vary.^5,35,36^ Hence, the results are addressed in association with the lubrication regimes.

Figure 3 and Table S4 in the Supporting Information further show that the negative effects of texturing on the COF diminish at higher sliding speeds and viscosities. It is expected that the aqueous solution with a higher viscosity will lead to form a thicker and steadier interfacial liquid film and reduce friction.^7,37,38^ Agreeing with the expectation, the result shows that the increase in the aqueous viscosity lowers the COF in most cases, regardless of the presence of pore textures and the sliding speed applied. Among the results shown in Table S4, only the COF values of untextured surfaces at relatively high speeds (1 and 2 mm/s) show no significant differences (*p* > 0.99) for the variation of the aqueous viscosity. Meanwhile, it should be noted that the COF values of untextured surfaces reach the lowest values amongst all the surfaces examined in this study. For example, the mean COF values of the untextured surfaces at 1 and 2 mm/s lie between 0.01 and 0.05, which are at least 4 to 460 times smaller than those of the pored surfaces with the same viscosity and sliding speed.

### 3.2 Effects on the Lubrication Regimes

**Figure 5:**
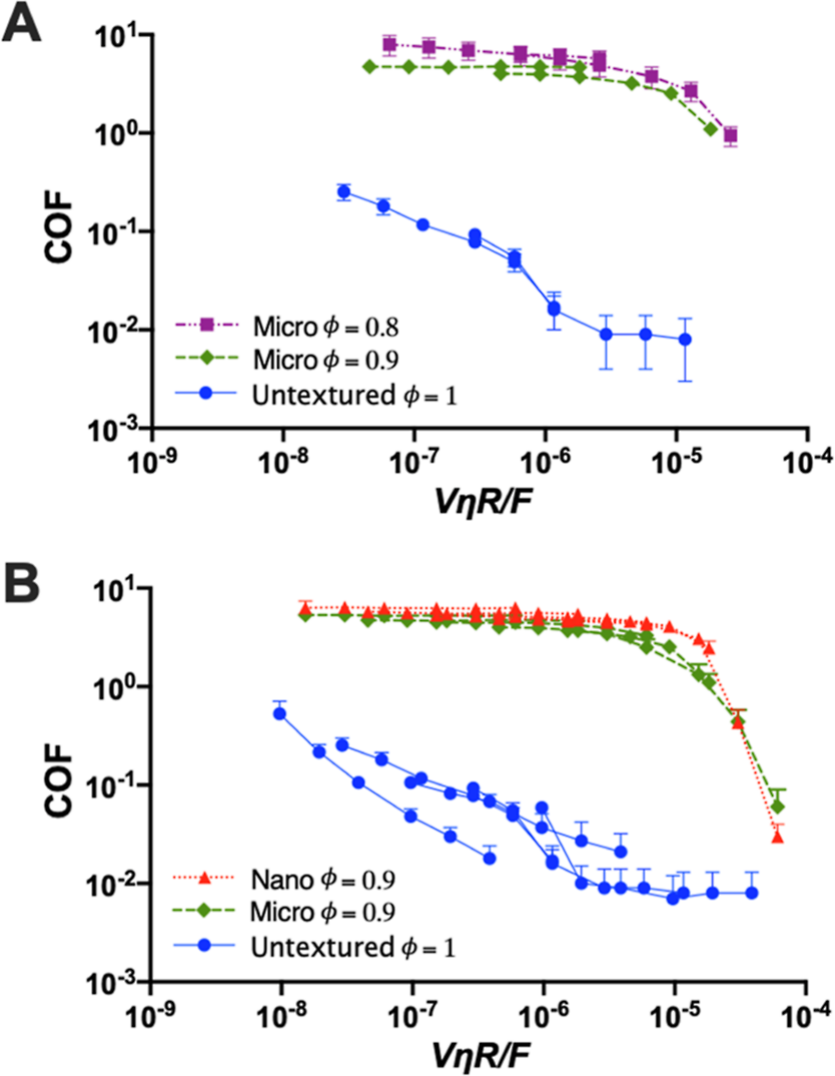
Stribeck curves. (A) Comparison of the micropored surfaces with the different solid area fractions (i.e., ϕ = 0.8 and 0.9) to the untextured surface with the viscosities of 3 and 30 mPa·s. (B) Comparison of the nano- and micropored surfaces to the same solid area fraction (ϕ = 0.9) to the untextured surface with viscosities from 1 to 100 mPa·s. *X*-axis is the modified Stribeck number = (sliding speed (*V*) × viscosity (η) × PDMS probe curvature (*R*))/load (*F*). (See Table S2 in the Supporting Information for the values of *F* for specimens with different ϕ.)

To address the effects of the textures on the COF values in association with lubrication regimes, the COF values are plotted in the form of Stribeck curves, as shown in Figure 5. The dimensionless Stribeck number takes sliding speed, aqueous viscosity, and applied load at the same time. The Stribeck curves (Figure 5) indicate that the untextured surface begins in the boundary lubrication regime and mostly stays within a mixed lubrication regime (combination of solid–solid contact and solid–liquid–solid contact). As the Stribeck number increases, the COF gradually decreases, indicating the establishment of a more continuous lubricant film between the contact surfaces. Yet, the decrease tends to stop at the Stribeck number around 10^–6^ with the COF value reaching around 0.01 and staying around 0.01 with higher Stribeck numbers (Figure 5B). This behavior suggests that the system is approaching a transition to a possible hydrodynamic lubrication regime where a continuous lubricant film separates the surfaces and minimizes solid–solid contact;^39,40^ however, the system has not yet fully reached this regime, and some degree of solid–liquid–solid contact may still be present.

In contrast, the Stribeck curves illustrate that the lubrication regime of the pored silicon surfaces mostly stays within a boundary lubrication regime (mostly solid–solid contact) with the COF value greater than 1 and then transitions to a mixed lubrication regime with the sharp decrease in the COF value at the Stribeck number around 10^–5^. The results suggest that the aqueous solution occupied in the pores could not help to provide the contact interface with the effective liquid film to reach a hydrodynamic lubrication regime. The texture’s edge effect discussed earlier should also prevent the pored surfaces from approaching the hydrodynamic lubrication regime. In other words, the pore textures artificially render the discontinuity of the constitution of a liquid film at the interface, causing the unstable interfacial contact (i.e., solid–solid contact with a heterogeneous interface caused by the pore textures) for the boundary lubrication regime, giving rise to high COF values. Previously, several works^5,41−45^ also showed that microtexturing could delay the transition of the lubrication regimes (e.g., with the extension of the boundary lubrication regime) due to the discontinuity of the liquid at the heterogeneous interface caused by the textures, leading to an increase in the COF value under the boundary and mixed lubrication regimes. The present results suggest such effects are still valid even with nanotexturing.

### 3.3 Effect of Material Stiffness and Hydrophilicity on Friction

In our previous work,^5^ we studied the tribological system consisting of a hard and hydrophilic spherical glass probe sliding against soft and hydrophobic PDMS substrates (untextured and micropored PDMS with ϕ = 0.8 and 0.9). In this study, the pair has been switched to mimic the soft tissue-hard implant interface by using a soft and hydrophobic spherical PDMS probe sliding against hard and hydrophilic silicon substrates with the same micropore textures. Both cases show that the pore textures result in an increase in friction compared to the untextured surfaces; both cases also show that even when the protein layers (i.e., reconstituted human whole saliva) were coated onto all the specimens, the frictional forces with the protein-coated pore-textured silicon substrates still had higher mean than those with the protein-coated untextured silicon substrates under relatively low sliding speeds and low aqueous viscosities (i.e., 1 mm/s and 1 mPa·s) (Figure S1 in the Supporting Information). Meanwhile, we note that the increase in friction is much greater in the present case. For example, under the contact pressure of 100 kPa, the sliding speed of 0.1 mm/s, and the aqueous viscosity of (1 mPa·s), the maximum increase in the mean COF value due to micropore textures in the former case of glass-probe-on-PDMS-substrate was less than 1.3 times, while both the untextured and textured PDMS surfaces pertain to a boundary lubrication regime.^5^ However, under the same tribological conditions in the present case of PDMS-probe-on-silicon-substrate texturing causes over 25 times increase because the untextured silicon surface pertains to a mixed boundary lubrication regime and the micropored silicon surfaces pertain to a boundary lubrication regime. The reason for this difference could be the difference in deformability of the texture under compression caused by the difference in stiffness of the textured material. PDMS is about 5 × 10^4^ softer than glass or silicon by Young’s modulus. The textures on PDMS can be easily pressed and flattened under the pressure of the spherical glass probe, whereas the textures on silicon would maintain their shape and depth when pressed against the PDMS pin. Thus, the texture’s edge effect is more pronounced and critical to causing friction in this study than in our previous study of the glass-probe-on-PDMS-substrate.

Hydrophobicity could also play an important role in tribology. Of note, the tribological properties of micropored hydrogel material (i.e., pHEMA, which is hydrophilic and slides against glass) were tested in our previous study.^9^ As opposed to the silicon and PDMS (hydrophobic) materials, it was found that the micropore textures could effectively reduce the friction. Unlike most of the solid materials (i.e., silicon and PDMS), the aqueous liquid is absorbed in the hydrogel material including bound water, intermediate water, and free water (total water content of about 40% for the pHEMA). For such a hydrogel material, the tribological properties could not effectively be understood with a classic Stribeck curve.^46−48^ Therefore, it is hard to directly compare the results of the micropored pHEMA to those of micropored silicon or PDMS from the perspective of the lubrication regimes using the Stribeck curve. Nonetheless, the uniqueness of the hydrogel material for the tribological properties can still be understood from the perspective of the water available at the interface. The free water from the hydrogel material or the surrounding aqueous area should always be available to lubricate the contact interface.

### 3.4 Limitations

Silicon wafers successfully mimic most of the hard biomaterials (i.e., metals and ceramics) due to its high stiffness and hydrophilicity. But PDMS possibly does not fully mimic all the different soft tissues of interest because of its hydrophobicity and fixed stiffness. Furthermore, *in vivo* the tissue–biomaterial interface could be lubricated with the help of extracellular fluid, which was mimicked in our study simply with a mixture of glycerol and Milli-Q water. Last but not least, we have only studied pore textures, and the texture edge might play a different role for pillar and line textures.

## 4 Conclusions

The tribology of a textured, hard biomaterial sliding against soft tissue was studied. Under physiologically relevant tribological conditions, both micropored and nanopored silicon surfaces paired with a PDMS probe increased friction, compared to the untextured surface. Since most cases with textured surfaces fall under a boundary lubrication regime in our study, the entrainment of an aqueous film at the interface did not significantly contribute to the lubrication between the two contact surfaces. Under a solid–solid contact between two tribo-paired objects (i.e., soft-probe-on-hard-substrate) with a large difference in material stiffness, the greater texture’s edge effect resulted in the larger friction. This study shows that the texture’s edge effect has two different aspects, i.e., texture edge length per unit area and pore diameter. In general, higher texture edge length per unit area increases the friction, and this aspect helps explain the effect of scale of texturing, i.e., the friction of nano- as compared to microtexturing. When the texture edge length per unit area remains the same, the pore patterns with a larger pore diameter further increase the friction due to the larger deformation of the soft material. Our results also revealed that the increase in friction from the texturing can be more pronounced in an aqueous environment with a relatively low viscosity and low sliding speeds than with a relatively high viscosity and high speeds; this negative effect from the texturing (i.e., increase in friction) cannot be reversed by the lubricious coating of protein layers under a relatively low viscosity and low sliding speed. Hence, great care needs to be taken when a micro- or nanotextured solid material is applied to an implant surface *in vivo*.

## Supporting Information Available

Mechanical properties of the PDMS tip and the silicon substrate; estimation of the applied loads and contact radii; and COF of protein-coated and uncoated silicon substrates with and without texturing (PDF)
