# Supplementary material for: Tribology of Pore-Textured Hard Surfaces under Physiological Conditions: Effects of Texture Scales
Source: Langmuir. 2023 May 1;39(19):6657–65. doi: 10.1021/acs.langmuir.2c03377 (PMC10193583; doi:10.1021/acs.langmuir.2c03377)
Supplement: Supplementary file 1 — la2c03377_si_001.pdf [file la2c03377_si_001.pdf]

SUPPORTING INFORMATION

Tribology of Pore Textured Hard Surfaces under Physiological Conditions: Effects of Texture Scales

Yiwen Xi[1,2,3], Chang-Hwan Choi[1], Robert Chang[1], Hans Jan Kaper[2,3], Prashant Kumar Sharma[2,3*]*

[1] Department of Mechanical Engineering, Stevens institute of Technology, Castle Point on Hudson, Hoboken, New Jersey 07030, United States

[2] University of Groningen and University Medical Center Groningen, Department of Biomedical Engineering-FB40, A. Deusinglaan 1, 9713AV Groningen, The Netherlands

[3] University of Groningen, University Medical Center Groningen, W.J. Kolff Institute for Biomedical Engineering and Materials Science-FB41, A. Deusinglaan 1, 9713 AV Groningen, The Netherlands

* Corresponding authors with equal contribution.

Table S1. Mechanical properties of PDMS tip and silicon substrate.

	**Upper Fixed Object: PDMS Probe**	**Lower Sliding Object: Silicon substrate**
**Radius of Curvature, *R* (mm)**	3.1	∞ (i.e., flat)
**Young's Modulus, *E* (MPa)**	2.6	1.3×10^5
**Poisson's Ratio, $\boldsymbol{\upsilon}$**	0.5	0.28

Table S2. Applied loads and contact radii for average contact pressure, P= 100 kPa, on the silicon surfaces with the varied solid area fraction.

**Solid Area Fraction, ϕ**	**Load, *F* (mN)**	**Contact Radius, r_{HC} (μm)**
**1** (Untextured)	16.0	221
**0.9** (Micro & Nano)	10.2	190
**0.8** (Micro)	7.2	169

Note: $P = \frac{F}{\pi r_{HC}^2 \cdot \phi} = \frac{F^{\frac{1}{3}}}{\pi\left(\frac{3}{4}\frac{R}{E^*}\right)^{\frac{2}{3}}\phi}$, where $\frac{1}{E^*} = \frac{1-\upsilon_1^2}{E_1} + \frac{1-\upsilon_2^2}{E_2}$

Table S3. Statistical significance of the effect of textures: The results of two-way ANOVA analysis comparing the COF values of two different surfaces under the same load, speed, and aqueous viscosity (NS: not significant; *: $p < 0.05$; **: $p < 0.001$; ***: $p < 0.005$; ****: $p < 0.0001$; N/A: not available).

<table>
<tr><th>Viscosity, η (mPa · s)</th><th colspan="6">1</th><th colspan="6">3</th><th colspan="6">10</th><th colspan="6">30</th><th colspan="6">100</th></tr>
<tr><th>Velocity, V (mm/s)</th><th>0.05</th><th>0.1</th><th>0.2</th><th>0.5</th><th>1</th><th>2</th><th>0.05</th><th>0.1</th><th>0.2</th><th>0.5</th><th>1</th><th>2</th><th>0.05</th><th>0.1</th><th>0.2</th><th>0.5</th><th>1</th><th>2</th><th>0.05</th><th>0.1</th><th>0.2</th><th>0.5</th><th>1</th><th>2</th><th>0.05</th><th>0.1</th><th>0.2</th><th>0.5</th><th>1</th><th>2</th></tr>
<tr><td>Nano $\Phi = 0.9$ vs. $\Phi = 1$</td><td colspan="6">****</td><td colspan="6">****</td><td colspan="6">****</td><td colspan="6">****</td><td colspan="5">****</td><td>NS</td></tr>
<tr><td>Micro $\Phi = 0.9$ vs. $\Phi = 1$</td><td colspan="6">****</td><td colspan="6">****</td><td colspan="6">****</td><td colspan="6">****</td><td colspan="5">****</td><td>*</td></tr>
<tr><td>Micro $\Phi = 0.8$ vs. $\Phi = 1$</td><td colspan="6">N/A</td><td colspan="6">****</td><td colspan="6">N/A</td><td colspan="6">****</td><td colspan="6">N/A</td></tr>
<tr><td>Micro $\Phi = 0.8$ vs. Micro $\Phi = 0.9$</td><td colspan="6">N/A</td><td colspan="6">****</td><td colspan="6">N/A</td><td colspan="6">****</td><td colspan="6">N/A</td></tr>
<tr><td>Nano $\Phi = 0.9$ vs. Micro $\Phi = 0.9$</td><td colspan="6">****</td><td colspan="6">****</td><td colspan="6">****</td><td colspan="6">****</td><td colspan="4">****</td><td>NS</td><td>NS</td></tr>
</table>

Table S4. Statistical significance of the effect of aqueous viscosity: The results of two-way ANOVA analysis comparing the COF values under different aqueous viscosities at the same sliding speed of each individual surface (NS: not significant; *: $p < 0.05$; **: $p < 0.001$; ***: $p < 0.005$; ****: $p < 0.0001$; N/A: not available).

<table>
<tr><th colspan="2">Surface type</th><th colspan="6">$\phi = 1$</th><th colspan="6">Nano $\phi = 0.9$</th><th colspan="6">Micro $\phi = 0.9$</th><th colspan="6">Micro $\phi = 0.8$</th></tr>
<tr><th>Viscosity, η (mPa·s)</th><th>Velocity, V (mm/s) / Difference ratio in viscosity</th><th>0.05</th><th>0.1</th><th>0.2</th><th>0.5</th><th>1</th><th>2</th><th>0.05</th><th>0.1</th><th>0.2</th><th>0.5</th><th>1</th><th>2</th><th>0.05</th><th>0.1</th><th>0.2</th><th>0.5</th><th>1</th><th>2</th><th>0.05</th><th>0.1</th><th>0.2</th><th>0.5</th><th>1</th><th>2</th></tr>
<tr><td>1 vs. 3</td><td>3</td><td colspan="2">****</td><td>NS</td><td>****</td><td colspan="2">NS</td><td colspan="6">****</td><td colspan="6">****</td><td colspan="6">N/A</td></tr>
<tr><td>3 vs. 10</td><td>~ 3</td><td colspan="4">****</td><td colspan="2">NS</td><td colspan="6">****</td><td colspan="6">****</td><td colspan="6">N/A</td></tr>
<tr><td>10 vs. 30</td><td>3</td><td colspan="3">****</td><td>*</td><td colspan="2">NS</td><td colspan="6">****</td><td colspan="6">****</td><td colspan="6">N/A</td></tr>
<tr><td>30 vs. 100</td><td>~ 3</td><td colspan="2">****</td><td colspan="4">NS</td><td colspan="6">****</td><td colspan="6">****</td><td colspan="6">N/A</td></tr>
<tr><td>1 vs. 10</td><td>10</td><td colspan="3">****</td><td colspan="3">NS</td><td colspan="6">****</td><td colspan="6">****</td><td colspan="6">N/A</td></tr>
<tr><td>3 vs. 30</td><td>10</td><td colspan="4">****</td><td colspan="2">NS</td><td colspan="6">****</td><td colspan="6">****</td><td colspan="6">****</td></tr>
</table>

**10 *vs.* 100**	10	****	*	**NS**	****	****	**N/A**
**1 *vs.* 30**	30	****		**NS**	****	****	**N/A**
**3 *vs.* 100**	~30	****		**NS**	****	****	**N/A**
**1 *vs.* 100**	100	****		**NS**	****	****	**N/A**

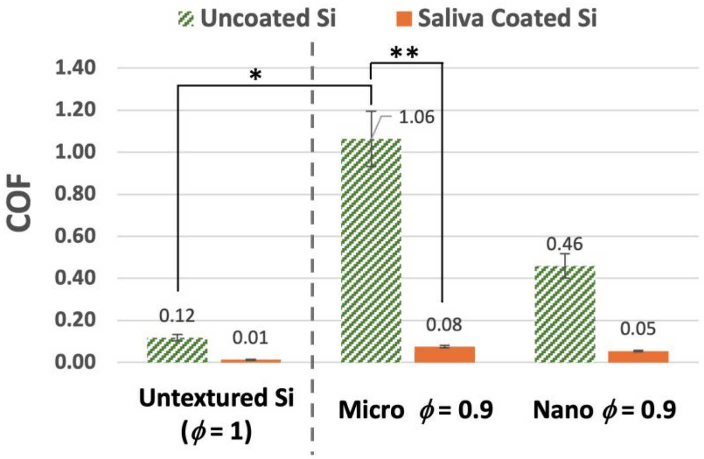

Figure S1. COF of protein-coated (i.e., reconstituted human whole saliva (RHWS)*) and uncoated silicon specimens with and without texturing at contact pressure of 100 kPa, sliding speed of 1 mm/s and aqueous viscosity of 1 mPa·s. Statistically significant difference (two-tailed *z*-tests): *: $p < 0.05$; **: $p < 0.001$. RHWS was collected from twenty healthy volunteers who were working in the Biomedical Engineering Department of the University Medical Center Groningen (UMCG) in 2016 spring under approval no. M09.069162 of the Medical Ethics Committee from the UMCG. RHWS were dissolved in adhesion buffer pH = 6.8 (1 mM $CaCl_2$, 2 mM KPi, 50 mM KCl and H_2O) at a concentration of 1.5 g/l. Adsorbed protein layers were obtained by dip-coating for 12 h at 4 °C.
